# Skeletal Myoblast Cells Enhance the Function of Transplanted Islets in Diabetic Mice

**DOI:** 10.1155/2024/5574968

**Published:** 2024-05-17

**Authors:** Takeshi Kado, Yoshito Tomimaru, Shogo Kobayashi, Akima Harada, Kazuki Sasaki, Yoshifumi Iwagami, Daisaku Yamada, Takehiro Noda, Hidenori Takahashi, Shunbun Kita, Iichiro Shimomura, Shigeru Miyagawa, Yuichiro Doki, Hidetoshi Eguchi

**Affiliations:** ^1^Department of Gastroenterological Surgery, Graduate School of Medicine, Osaka University, Osaka, Japan; ^2^Department of Cardiovascular Surgery, Graduate School of Medicine, Osaka University, Osaka, Japan; ^3^Department of Metabolic Medicine, Graduate School of Medicine, Osaka University, Osaka, Japan; ^4^Department of Adipose Management, Graduate School of Medicine, Osaka University, Osaka, Japan

## Abstract

Islet transplantation (ITx) is an established and safe alternative to pancreas transplantation for type 1 diabetes mellitus (T1DM) patients. However, most ITx recipients lose insulin independence by 3 years after ITx due to early graft loss, such that multiple donors are required to achieve insulin independence. In the present study, we investigated whether skeletal myoblast cells could be beneficial for promoting angiogenesis and maintaining the differentiated phenotypes of islets. In vitro experiments showed that the myoblast cells secreted angiogenesis-related cytokines (vascular endothelial growth factor (VEGF), hepatocyte growth factor (HGF), and stromal-derived factor-1*α* (SDF-1*α*)), contributed to maintenance of differentiated islet phenotypes, and enhanced islet cell insulin secretion capacity. To verify these findings in vivo, we transplanted islets alone or with myoblast cells under the kidney capsule of streptozotocin-induced diabetic mice. Compared with islets alone, the group bearing islets with myoblast cells had a significantly lower average blood glucose level. Histological examination revealed that transplants with islets plus myoblast cells were associated with a significantly larger insulin-positive area and significantly higher number of CD31-positive microvessels compared to islets alone. Furthermore, islets cotransplanted with myoblast cells showed JAK-STAT signaling activation. Our results suggest two possible mechanisms underlying enhancement of islet graft function with myoblast cells cotransplantation: “indirect effects” mediated by angiogenesis and “direct effects” of myoblast cells on islets via the JAK-STAT cascade. Overall, these findings suggest that skeletal myoblast cells enhance the function of transplanted islets, implying clinical potential for a novel ITx procedure involving myoblast cells for patients with diabetes.

## 1. Introduction

Type 1 diabetes mellitus (T1DM) is a disease that carries a poor prognosis. For patients with T1DM, islet transplantation (ITx) is a promising treatment, considered a safe and effective alternative to pancreas transplantation [1–4]. Most ITx recipients initially experience freedom from life-threatening hypoglycemic events; however, they commonly lose insulin independence by 3 years after ITx, due to early graft loss [5, 6]. Therefore, multiple donors are often required to achieve the desired clinical effects [4].

Several possible solutions have been proposed for this issue with ITx. Notably, it would help if the islets had rapidly established vascular networks and enhanced insulin secretion capacity. This could potentially be accomplished by generating vascularized islets from dissociated cell suspensions via coculturing with mesenchymal stem cells and human umbilical cord-derived endothelial cells [7]. However, no clinically applicable methods have yet been developed.

To help resolve this issue, in our present study, we investigated the possibility that skeletal myoblast cells could help promote angiogenesis and the maintenance of differentiated phenotypes of islets. Myoblast cells have been reported to facilitate the repair of injured skeletal muscle, and sheets produced by skeletal myoblast cells have been studied in several fields of organ regeneration [8–11]. For example, in cardiac regeneration, an autologous skeletal myoblast cell sheet was found to functionally prevent deterioration of impaired myocardium in an animal model of infarction [12, 13]. Moreover, the effect of sheet transplantation on impaired cardiac function has been confirmed in humans [14]. One mechanism underlying these effects of skeletal myoblast cell sheets is a paracrine effect associated with growth factors, including vascular endothelial growth factor (VEGF), hepatocyte growth factor (HGF), and stromal-derived factor-1*α* (SDF-1*α*) [11, 12, 15, 16]. These growth factors are associated with angiogenesis [17–20]. Moreover, García-Ocaña et al. demonstrated that HGF gene transfer increases islet number, islet size, and overall islet mass in rodents [21, 22].

These previous findings suggest that myoblast cells could potentially enhance survival and function of transplanted islets in ITx. If this possibility is verified, these cells could be helpful for overcoming the clinical problems associated with ITx. In the present study, we focused on cotransplantation of myoblast cells with islets as a new therapeutic option in ITx for T1DM patients. We investigated the efficacy of myoblast cell transplantation for ITx in both in vitro and in vivo models.

## 2. Materials and Methods

### 2.1. Animals and Ethics Statement

For islet donors, we used 10- to 12-week-old Jcl:ICR male mice. For diabetic recipients, we used 8–10-week-old Jcl:ICR mice. All mice were purchased from Clea Japan (Tokyo, Japan) and were housed under specific pathogen-free conditions. The experimental protocol for the animal experiments was approved by the Ethics Review Committee for Animal Experimentation of Osaka University (approval number 04-084-000). All animal experiments were conducted in accordance with the Institutional Animal Care and Use Committee Guidelines of Osaka University.

### 2.2. Islet Isolation

Mice were anesthetized with isoflurane, and an abdominal incision was made. After clamping the duodenal papilla, the common bile duct was cannulated using a 30-gauge needle and perfused with Hank's balanced salt solution (Nacalai Tesque, Kyoto, Japan) containing 1 mg/mL collagenase V (Sigma-Aldrich, St. Louis, MO, USA). The pancreas was dissected and the sample digested in 1 mg/mL collagenase V by shaking for 18 min at 37°C. After incubation, digestion was stopped by the addition of 20 mL of RPMI 1640 medium (Nacalai Tesque) containing 10% fetal bovine serum (FBS). Centrifugation (300 × *g* for 1 min at 4°C) was performed, and the pellet was suspended in 13 mL of Histopaque-1077 (Sigma-Aldrich). Next, 12 mL of RPMI 1640 was gently poured onto the suspension to form a bilayer. Centrifugation (1500 × *g* for 24 min at 4°C) was performed, and we confirmed the presence of the islets between the two layers. Islets were hand-picked using a 1-mL pipette and incubated overnight (37°C, 5% CO_2_) in Dulbecco's modified Eagle medium (DMEM) (Nacalai Tesque) containing 10% FBS, 100 mg/mL streptomycin, and 100 units/mL penicillin in preparation for use.

### 2.3. Culture of Mouse Skeletal Myoblast Cells

Myoblast cells derived from ICR mice were purchased from Cosmo Bio (Tokyo, Japan). These cells were cultured (37°C, 5% CO_2_) in DMEM supplemented with 20% FBS and 1% penicillin-streptomycin [23]. Cells were subcultured using 0.05% trypsin and 0.02% EDTA in phosphate-buffered saline (PBS) (Nacalai Tesque). Myoblast cells at passages 3–5 were used for the experiments.

### 2.4. Measurement of Cytokine Secretion

ELISA was performed to measure cytokine production levels in the culture media from islets cultured alone and from islets cocultured with myoblast cells. Myoblast cells were disseminated at 1.0 × 10^5^ per well onto 24-well plates (Corning Inc, Corning, NY, USA). Then, 30 islets were seeded into 24-well culture inserts with 0.4-*μ*m pore membranes (Greiner Bio-One, Kremsmünster, Austria), followed by incubation at 37°C for 24 and 72 h. After incubation, the culture supernatants were collected from each well and analyzed. VEGF, HGF, SDF-1*α*, and interleukin-6 (IL-6) concentrations were measured using the Mouse VEGF ELISA kit (Abcam, Cambridge, UK), Mouse HGF ELISA kit, Mouse SDF-1*α* ELISA kit, and Mouse IL-6 ELISA kit (R&D Systems, Minneapolis, MN, USA), according to the manufacturers' protocols.

### 2.5. Glucose-Stimulated Insulin Secretion (GSIS)

For GSIS, testing was performed as follows. First, 30 islets were seeded into culture inserts (Corning Inc). Then, 600 *μ*L of 3.3 mM glucose solution was added to each well of a 24-well plate, and each insert was soaked in a well for 1 h. Next, each insert was soaked again in a different well containing a new 600-*μ*L aliquot of the 3.3 mM glucose solution. After 1 h, the insert was removed, and the remaining liquid in the well was sampled (low glucose sample). The removed insert was immediately transferred to a new well containing 600 *μ*L of 20 mM glucose solution and soaked for 1 h. Then, the insert was removed, and the remaining solution in the well was sampled (high glucose sample). In the low glucose and high glucose samples, the insulin concentrations were quantified using the Mouse Insulin ELISA kit (Mercodia, Uppsala, Sweden). Insulin secretion levels were standardized according to protein level of islets. Then, the stimulation index, defined as the ratio of released insulin at conditions of high versus low glucose stimulation, was calculated.

### 2.6. Western Blot Analysis

After 24 h of coculture, total proteins were extracted from islets using RIPA buffer (Thermo Fisher Scientific, Waltham, MA, USA) containing protease and phosphatase inhibitor. Homogenates were purified by centrifugation at 12,000 × *g* at 4°C for 12 min, and protein concentrations were determined using a bicinchoninic acid protein assay (Thermo Fisher Scientific). Equal amounts of protein extract were separated by sodium dodecyl sulfate polyacrylamide gel electrophoresis on 12% Tris-HCl gels (Bio-Rad Laboratories Inc, Hercules, CA, USA). The separated proteins were then transferred to polyvinylidene difluoride membranes (Bio-Rad Laboratories Inc) and incubated for 1 h with the following primary antibodies: anti-signal transducer and activator of transcription (STAT) 3 (Cell Signaling Technology, Danvers, MA, USA), anti-phosphorylated STAT3 (pSTAT3) (Cell Signaling Technology), and anti-*β* actin (Sigma-Aldrich). Next, the membranes were incubated with horseradish peroxidase–linked anti-rabbit IgG (GE Healthcare, Buckinghamshire, UK) at room temperature for 1 h. The antigen–antibody complex was detected using the ECL Prime Western Blotting Detection kit (GE Healthcare).

### 2.7. Reverse Transcription Polymerase Chain Reaction (RT-PCR)

After 24 h of coculture, total RNA was isolated from islets using the RNeasy Mini Kit (Qiagen, Hilden, Germany). The RNA integrity and concentration were measured using NanoDrop Lite (Thermo Fisher Scientific). RNA was reverse-transcribed and subjected to quantitative RT-PCR as previously described [24]. For quantitative PCR, complementary DNA was synthesized using the Reverse Transcription System (Promega, Madison, WI, USA). Procedures were performed in triplicate with the VilA7 Software (Thermo Fisher Scientific), based on the Thunderbird SYBR qPCR Mix (Toyobo Co, Ltd, Osaka, Japan). The results were normalized to the mouse *gapdh* housekeeping gene and presented as the fold difference over the detectable Ct value, calculated using the *ΔΔ*Ct method. Table S1 presents the primer sequences for quantitative reverse transcription.

### 2.8. Diabetic Mice and ITx

For diabetic recipients, we used 8–10-week-old male Jcl:ICR mice. At 7 days before transplantation, diabetes was induced using streptozotocin (STZ) (Sigma-Aldrich) in PBS, administered intravenously at 160 mg/kg [25]. Diabetes was defined as a nonfasting blood glucose level > 400 mg/dL detected twice consecutively after STZ injection. For ITx experiments, we prepared pellets of 200 islets and pellets of 200 islets with 1 × 10^6^ myoblast cells without prior coculture. Each type of graft was implanted under the left kidney capsule in a recipient mouse, as previously described [26]. As a control, a sham operation without graft transplantation was performed. Before the procedure, recipient mice were randomly divided into the following three groups: islet alone group (*n* = 8), islet + myoblast group (*n* = 8), and sham group (*n* = 6). After transplantation, nonfasting blood glucose and body weight were measured every other day for monitoring of islet graft function. Blood glucose level was measured using a blood glucose meter (Medisafe FIT; TERUMO, Tokyo, Japan) in blood from a tail pinprick. Euglycemia was defined as a nonfasting blood glucose level < 200 mg/dL on two consecutive days. At 30-day posttransplantation, engrafted kidneys were dissected out in a survival surgery, and the blood glucose level was further monitored. After endpoint monitoring, the mice were euthanized following an approved protocol.

### 2.9. Glucose Tolerance Test

To evaluate glucose responsiveness, an intraperitoneal glucose tolerance test (IPGTT) was performed 29 days after transplantation. Briefly, after an overnight fast, mice were intraperitoneally injected with 2.0 g/kg glucose in 0.9% NaCl, and then, blood glucose was measured at 0, 30, 60, 90, and 120 min. The blood glucose area under the curve (AUC) was calculated and compared between transplant groups [25].

### 2.10. BrdU Incorporation Assay

Before the procedure, recipient mice were randomly divided into the following two groups: islet alone group (*n* = 5) and islet + myoblast group (*n* = 5). Islets were transplanted under the left kidney capsule using the method described above. Bromodeoxyuridine (BrdU) (Nacalai Tesque) (50 mg/kg body weight) was intraperitoneally administered for four consecutive days, starting on the third day after transplantation. At 4 h after the last BrdU injection, the kidneys were dissected. We performed immunostaining of insulin and BrdU using the appropriate antibodies. The proportion of insulin and BrdU double-positive cells among the total insulin-positive cells was calculated.

### 2.11. RNA-Sequence

After 24 h of coculture, total RNA was isolated from islets using the RNeasy Mini Kit (Qiagen). Raw RNA sequence data were obtained using an Illumina NovaSeq™ 6000 machine. After acquiring data regarding the raw count and fragments per kilobase of exon per million, we obtained mapped reads. For each RNA, we calculated the fold change (mean of each RNA in the islet + myoblast group/mean of each RNA in the islet alone group) and *p* values, which were used as a filter to identify RNAs having a fold change of ≥ 1.2 or ≤ −1.2 and a *p* value of < 0.05. Enriched pathway analyses were performed using Gene Ontology biological process.

### 2.12. Immunohistochemical Assessment

For histological examination, removed kidneys were fixed with 10% formalin and embedded in paraffin. We used 4.0-*μ*m sections for immunofluorescence staining. After deparaffinization of the slides, antigens were retrieved using citrate buffer (pH 6). Next, the slides were blocked with 1% bovine serum albumin for 1 h at room temperature and then incubated overnight at 4°C with primary guinea pig anti-human insulin (ready to use, IR002; Dako-Agilent, Santa Clara, CA, USA), rabbit anti-CD31 (1:200, ab28364; Abcam), mouse anti-human smooth muscle actin (*α*-SMA) (1:100, M0851; Dako-Agilent), rabbit anti-phospho-Stat3 (1:200, Tyr705; Cell Signaling Technology), rat anti-BrdU (1:250, ab6326; Abcam), and rabbit anti-desmin (1:200, ab15200; Abcam). The following secondary antibodies were used: goat anti-guinea pig IgG (H + L), Alexa Fluor 488 (1:400, A11073; Invitrogen, Paisley, UK), goat anti-mouse IgG (H + L), Alexa Fluor 546 (1:200, A11003; Invitrogen), anti-rat IgG (H + L), Alexa Fluor 647 (1:400, 4418S; Cell Signaling Technology), and anti-rabbit IgG, Alexa Fluor 647 (1:400, 4414S; Cell Signaling Technology). Slides were incubated with secondary antibodies for 1 h at room temperature and mounted using ProLong Glass Antifade with NucBlue (Invitrogen). The mounted slides were evaluated under a fluorescence microscope (BZ-X700; Keyence, Osaka, Japan). Quantification for statistical evaluation was performed using a BZ-X Analyzer (Keyence).

### 2.13. Statistical Analysis

Statistical analyses were performed using JMP Pro 17.1.0 (SAS Institute Inc, Cary, NC, USA). All data are expressed as mean ± standard error of the mean. The means of continuous numerical variables were compared using Student's *t*-test for two variables. Statistical analyses of blood glucose, body weight gain rate, blood glucose change in the IPGTT, AUC for IPGTT, and insulin concentration were carried out with two-way analysis of variance (ANOVA) for repeated measures and Dunnett's multiple comparison. The percentage of mice with euglycemia after transplantation was calculated using the Kaplan–Meier method, and the log-rank test was used to evaluate the significance of differences. As noted, euglycemia was defined as two consecutive days of a nonfasting blood glucose level < 200 mg/dL. A *p* value of < 0.05 was considered statistically significant.

## 3. Results

### 3.1. Secretion Capacity of Myoblast Cells Cocultured With Islets

**Figure 1:**
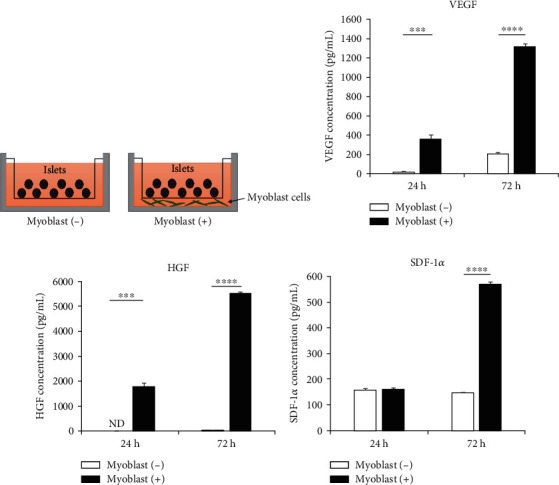
Secretion of angiogenesis-related cytokines by myoblast cells cocultured with islets. (a) Scheme for cell culture using culture inserts to investigate the cytokines secreted by the myoblasts and their effects on the islets. Concentrations of angiogenesis-related cytokines—(b) VEGF, (c) HGF, and (d) SDF-1*α*—in the supernatants of myoblast cells cultured with islets, after 24 and 72 h of culture, measured with ELISA (*n* = 3). Values were compared using Student's *t*-test. Data are presented as mean ± SEM. ^∗∗∗^*p* < 0.001 and ^∗∗∗∗^*p* < 0.0001. ELISA, enzyme-linked immunosorbent assay; HGF, hepatocyte growth factor; ND, not detected; SDF-1*α*, stromal-derived factor-1*α*; SEM, standard error of the mean; VEGF, vascular endothelial growth factor.

We first examined the cytokine secretion capacity of the myoblast cells cocultured with islets under in vitro culture conditions using tissue culture inserts (Figure 1(a)). Under the coculture conditions with islets, the myoblast cells were confirmed to secrete VEGF, HGF, and SDF-1*α* (Figures 1(b)–1(d)).

### 3.2. In Vitro Effects of Myoblast Cells on Insulin Secretion of Islets

**Figure 2:**
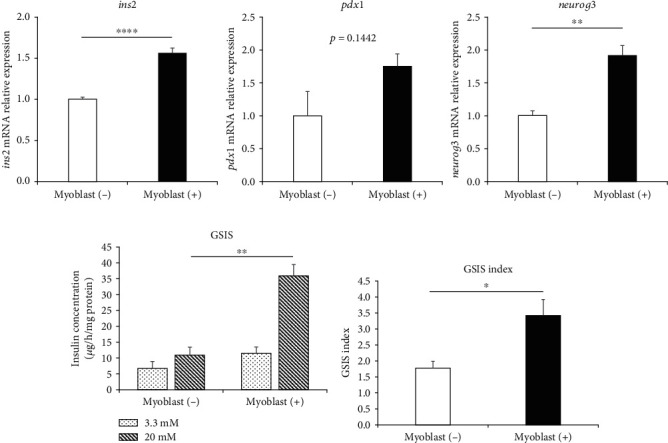
Enhanced function of islets cocultured with myoblast cells. (a) Expressions of genes associated with the maintenance of differentiated phenotypes of islets—*ins2* (left panel), *pdx1* (middle panel), *neurog3* (right panel)—in the islets cultured with and without myoblast cells, analyzed using RT-PCR. Data were normalized to the mouse housekeeping gene *gapdh*. Data are presented as fold changes compared with the values from the myoblast (−) group (*n* = 3). (b) Insulin secretion by islets cultured with myoblasts (right) and without myoblasts (left), evaluated by the GSIS assay, after 24 h of cococulture (*n* = 3). (c) GSIS index of islets cultured with myoblasts (right) and without myoblasts (left), determined as the concentration of secreted insulin in a 20 mM glucose solution divided by the concentration of secreted insulin in a 3.3 mM glucose solution. Values were compared using Student's *t*-test. Data are presented as the mean ± SEM. ^∗^*p* < 0.05, ^∗∗^*p* < 0.01, and ^∗∗∗∗^*p* < 0.0001. 3.3 mM, insulin secretion in 3.3 mM glucose solution; 20 mM, insulin secretion in 20 mM glucose solution; gapdh, glyceraldehyde-3-phosphate dehydrogenase; GSIS, glucose-stimulated insulin secretion; mRNA, messenger RNA; RT-PCR, reverse transcription polymerase chain reaction; SEM, standard error of the mean.

Next, we examined whether the myoblast cells enhanced the insulin secreting capacity of the islets in the in vitro experiment. After 24 h of coculture of islets with myoblast cells, the islets were assessed for the expression of genes involved in the maintenance of differentiated phenotypes (e.g., *ins2*, *pdx1*, and *neurog3*), using RT-PCR analysis. Although it was not significant, the expression level of *pdx1* mRNA was higher among islets cocultured with myoblast cells (myoblast (+) group), compared to the islets cultured without myoblast cells (myoblast (−) group). Additionally, *ins2* and *neurog3* mRNA expression levels were significantly higher among islets of the myoblast (+) group, compared to islets in the myoblast (−) group (Figure 2(a)). We also evaluated islet insulin secretion in both groups after 24 h of coculture. In the GSIS assay, islets in the myoblast (+) group released significantly more insulin, compared with islets in the myoblast (−) group (Figure 2(b)), and the stimulation index of islets in the myoblast (+) group was significantly higher, compared to among islets of the myoblast (−) group (Figure 2(c)). These in vitro results suggested that the myoblast cells contributed to the maintenance of differentiated phenotypes and enhanced islet insulin secretion capacity.

### 3.3. Enhanced Islet Graft Function by Myoblast Cell Cotransplantation in Diabetic Mice

**Figure 3:**
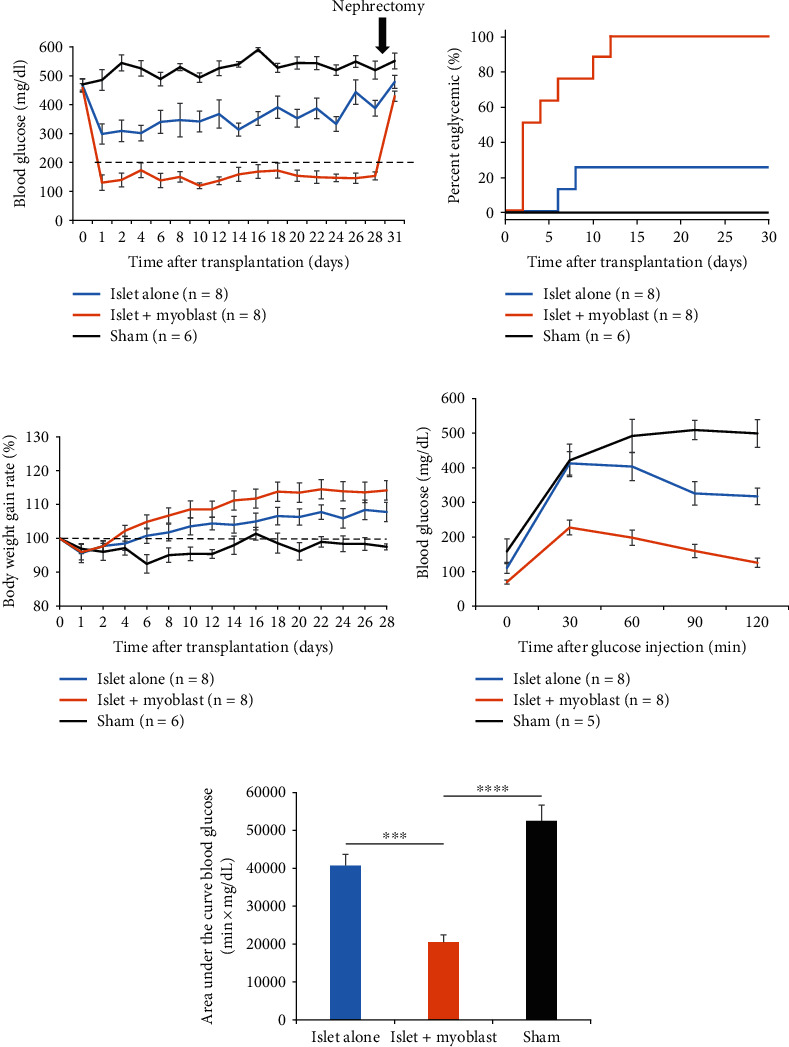
Effect of myoblast cell cotransplantation with islets on blood glucose level in diabetic mice. (a) The average nonfasting blood glucose levels after transplantation in the islet alone group (blue, *n* = 8), islet + myoblast group (orange, *n* = 8), and sham group (black, *n* = 6). At 30 days after transplantation, the kidney was removed (arrow). (b) Proportions of mice with euglycemia after transplantation in the islet alone group (blue, *n* = 8), islet + myoblast group (orange, *n* = 8), and sham group (black, *n* = 6). (c) The average body weight gain rate in the islet alone group (blue, *n* = 8), islet + myoblast group (orange, *n* = 8), and sham group (black, *n* = 6). (d) A IPGTT was performed 29 days after transplantation. The average blood glucose levels at 0, 30, 60, and 120 min after the glucose injection are shown for the islet alone group (blue, *n* = 8), islet + myoblast group (orange, *n* = 8), and sham group (black, *n* = 5). (e) The AUC for IPGTT in the islet alone group (white), islet + myoblast group (black), and sham group (gray). Values were compared using a repeated measures two-way ANOVA and Dunnett's multiple comparison. Data are presented as the mean ± SEM. ^∗∗∗^*p* < 0.001 and ^∗∗∗∗^*p* < 0.0001. ANOVA, analysis of variance; AUC, area under the curve; IPGTT, intraperitoneal glucose tolerance test; SEM, standard error of the mean.

The in vitro results led us to investigate whether myoblast cells would enhance islet graft function in diabetic mice. Islets alone or islets with myoblast cells were transplanted under the kidney capsule in STZ-induced diabetic mice, and blood glucose levels were measured after transplantation (Figure 3(a) and Figure S1). First, we confirmed that the average blood glucose level in the islet alone group significantly declined after transplantation compared to that in the sham group. The average blood glucose level in the islet + myoblast group remained below 180 mg/dL until graft removal. In the islet + myoblast group, the average blood glucose level significantly declined starting on the first day after transplantation and remained significantly lower compared to that in the islet alone group (*p* < 0.0001) and the sham group (*p* < 0.0001), until 28 days after transplantation (Figure 3(a)). Upon graft removal at 30 days after transplantation, all mice in the islet + myoblast group and in the islet alone group exhibited reelevated blood glucose levels, verifying that the reduced blood glucose levels were dependent on the transplanted graft in these groups. The proportion of mice with euglycemia after transplantation was significantly higher in the islet + myoblast group compared to that in the islet alone group (100% vs. 25%; *p* = 0.0010) and the sham group (100% vs. 0%; *p* = 0.0002) (Figure 3(b)). The rate of body weight gain in the islet + myoblast group was greater than in the islet alone group, but this difference was not significant (*p* = 0.2010), indicating no obvious adverse effect of myoblast cell cotransplantation (Figure 3(c)).

To further assess the function of transplanted islet grafts, we performed an IPGTT at 29 days after transplantation. Compared to that in the islet alone group, islet + myoblast transplantation significantly prevented elevation of blood glucose at 30 min (*p* = 0.0018), 60 min (*p* = 0.0018), 90 min (*p* = 0.0012), and 120 min (*p* < 0.0001) after glucose infusion (Figure 3(d)). AUC values for IPGTT were significantly lower in the islet + myoblast group than in the islet alone group (*p* = 0.0002) and in the sham group (*p* < 0.0001), validating the IPGTT results (Figure 3(e)). These findings also implied that enhanced islet graft function was responsive to glucose level, and overall, these data suggested that myoblast cells enhanced the function of the transplanted islet graft in diabetic mice.

### 3.4. Cotransplantation of Myoblast Cells With Islets Yields More Engrafted Islets and Promoted Angiogenesis in the Graft

**Figure 4:**
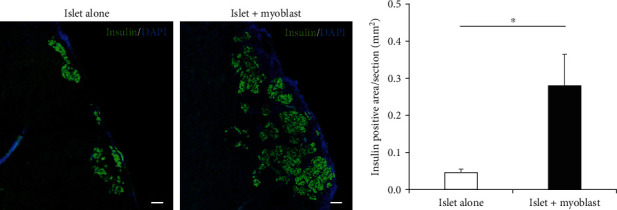
Histology of transplanted grafts with and without cotransplantation of myoblast cells. (a) Histology of engrafted islets in the islet alone group (left) and islet + myoblast group (right) at 30 days after transplantation. Green, insulin; blue, DAPI. (b) Area of engrafted islets under the kidney capsule from the islet alone group (white, *n* = 6) and islet + myoblast group (black, *n* = 6). Values were compared using Student's *t*-test. Data are presented as the mean ± SEM. ^∗^*p* < 0.05. Scale bars: 100 *μ*m. DAPI, 406-diamidino-2-phenylindole; SEM, standard error of the mean.

To identify possible mechanisms underlying the observed enhancement of transplanted islet graft function by myoblast cells, we histologically assessed the engrafted tissue removed from under the kidney capsule. Histological examination revealed that the islet + myoblast group exhibited a significantly larger insulin-positive area of the removed engrafted tissue compared to that in the islet alone group (Figures 4(a) and 4(b)).

**Figure 5:**
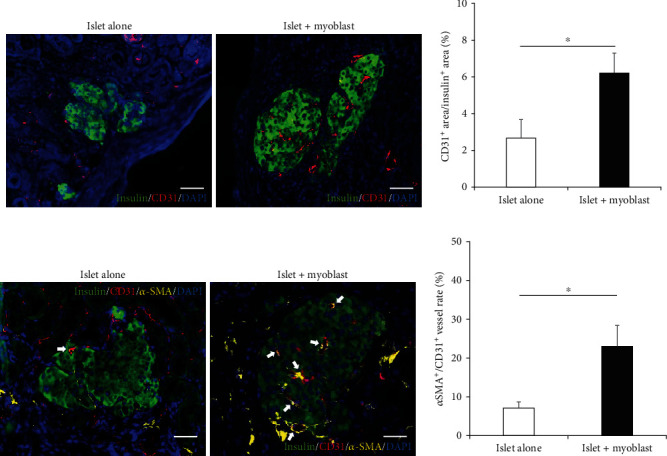
Histology of vascularization in transplanted islets with and without cotransplantation of myoblast cells. (a) Histology of the engrafted islets from the islet alone group (left) and islet + myoblast group (right) at 30 days after transplantation. Green, insulin; red, CD31; blue, DAPI. (b) Vessel area in the engrafted islets from the islet alone group (white, *n* = 6) and islet + myoblast group (black, *n* = 6). (c) Histology of engrafted islets from the islet alone group (left) and islet + myoblast group (right) at 30 days after transplantation. White arrows indicate CD31^+^/*α*-SMA^+^ vessels. Green, insulin; red, CD31; yellow, *α*-SMA; blue, DAPI. (d) CD31^+^/*α*-SMA^+^ vessel area in the engrafted islets from the islet alone group (white, *n* = 6) and islet + myoblast group (black, *n* = 6). Values were compared using Student's *t*-test. Data are presented as the mean ± SEM. ^∗^*p* < 0.05, *n* = 6, scale bars: 50 *μ*m. DAPI, 406-diamidino-2-phenylindole; SEM, standard error of the mean; SMA, smooth muscle actin.

Focusing on angiogenesis-related cytokine production by the myoblast cells, we evaluated vascularization to clarify how the myoblast cells affected angiogenesis. We confirmed that CD31-positive microvessels existed inside and around the engrafted islets from both the islet alone group and the islet + myoblast group, at 30 days posttransplantation. Comparison revealed a significantly higher number of CD31-positive microvessels in the islet + myoblast group, compared to the islet alone group (*p* = 0.0376) (Figures 5(a) and 5(b)). We further investigated the presence of CD31 and *α*-SMA double-positive vessels, which is an indicator of mature vessels [27]. The rate of *α*-SMA^+^/CD31^+^ vessels inside and around the engrafted islets was significantly higher in the islet + myoblast group than in the islet alone group (*p* = 0.0180) (Figures 5(c) and 5(d)). These data suggested that the myoblast cells promoted angiogenesis. Overall, the grafts in the islet + myoblast group had more engrafted islets and angiogenesis, which may have resulted in enhanced islet graft function.

### 3.5. JAK-STAT Signaling as a Mechanism Underlying the Enhanced Function of Islets In Vitro Cocultured With Myoblast Cells

To further investigate the mechanism behind the more engrafted islets in the graft following cotransplantation of myoblast cells with islets, we performed RNA sequencing to comprehensively analyze islet gene expression after 24 h of coculture with myoblast cells. Enriched pathway analyses revealed upregulation of the JAK-STAT cascade in the islets cocultured with myoblast cells (Figure S2).

**Figure 6:**
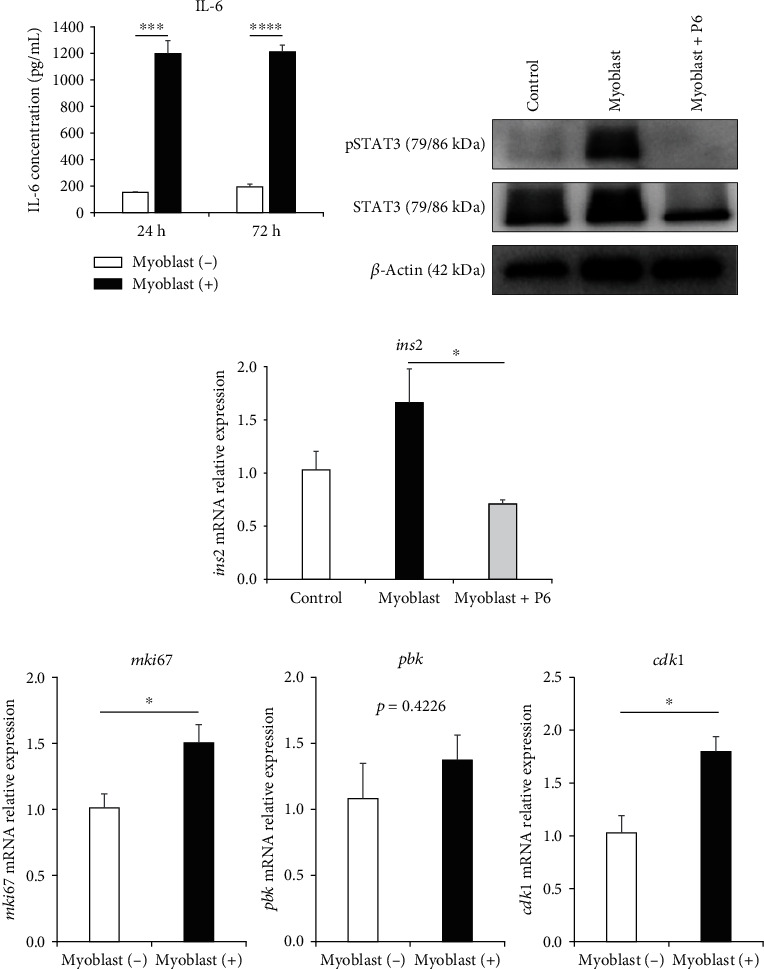
JAK-STAT signal activation in islets cocultured in vitro with myoblast cells. (a) IL-6 concentrations in supernatants after 24 and 72 h of culturing islets alone (white) or islets + myoblast cells (black), measured by ELISA (*n* = 3). (b) Western blot analysis showed expression of STAT3 and pSTAT3 in islets after 24 h of culture. (c) The *ins2* mRNA expression levels among islets (control group), islets cultured with myoblast cells (myoblast group), and islets cultured with myoblast cells + P6 (myoblast + P6 group), analyzed using RT-PCR. Data were normalized to the mouse housekeeping gene *gapdh* and expressed as fold changes compared with values from the control group (*n* = 3). (d) Expressions of genes associated with cell proliferation and the cell cycle—*mki67* (left panel), *pbk* (middle panel), and *cdk1* (right panel)—from islets cultured with or without myoblast cells, analyzed using RT-PCR. Data were normalized to the mouse housekeeping gene *gapdh* and expressed as fold changes compared with values from the myoblast (−) group (*n* = 3). Values were compared using Student's *t*-test for two variables and repeated measures two-way ANOVA and Dunnett's multiple comparison for three variables. Data are presented as the mean ± SEM. ^∗^*p* < 0.05, ^∗∗∗^*p* < 0.001, and ^∗∗∗∗^*p* < 0.0001. ANOVA, analysis of variance; gapdh, glyceraldehyde-3-phosphate dehydrogenase; IL-6, interleukin-6; P6, Pyridone 6; pSTAT3, phosphorylated signal transducer and activator of transcription 3; RT-PCR, quantitative reverse transcription polymerase chain reaction; SEM, standard error of the mean; STAT3, signal transducer and activator of transcription 3.

STAT was originally identified as a downstream mediator linking cell surface receptors to the nucleus in response to IL-6 family cytokines, and STAT3 in particular has been described as an important regulator of islet survival and function [28, 29]. Based on these previous reports, we first assessed IL-6 secretion from islets cocultured with myoblast cells. We found increased IL-6 secretion in the islets cocultured with myoblast cells, compared to the islets alone (Figure 6(a)). Next, based on a report that STAT3 can be phosphorylated in response to IL-6 [28], we examined STAT3 phosphorylation status in the islets. As expected, pSTAT3 was more strongly expressed in islets cocultured with myoblast cells, compared with islets cultured alone (Figure 6(b)).

To confirm whether STAT3 phosphorylation occurred through JAK activation, we then added Pyridone 6 (P6), a high-affinity competitive global inhibitor of JAK kinases, in this in vitro experiment [30]. We found that pSTAT3 expression was weakened in the cells incubated with 100 nM P6 for 24 h, which suggested that the enhanced pSTAT3 expression in islets cocultured with myoblast cells was mediated by JAK-STAT signaling (Figure 6(b)). Furthermore, the addition of P6 weakened the *ins2* mRNA expression level in islets when compared to islets cocultured with myoblast cells (Figure 6(c)). Based on a previous report describing STAT3 as a cell cycle modulator of islets [31], we also performed RT-PCR to examine expression of genes involved in cell proliferation and the cell cycle, including *mki67*, *pbk*, and *cdk1*. As expected, mRNA expression levels of these genes were higher among islets in the myoblast (+) group, compared to islets in the myoblast (−) group, although some of these differences were nonsignificant (Figure 6(d)). These results suggested that JAK-STAT signaling mediated the enhanced islet graft function upon the transplantation of myoblast cells with islets.

### 3.6. JAK-STAT Signal Activation in In Vivo Cotransplanted Islets With Myoblast Cells

**Figure 7:**
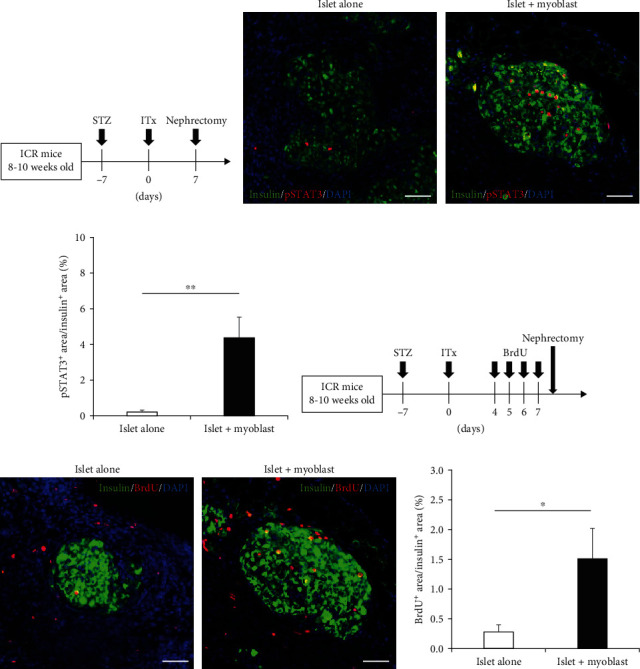
JAK-STAT signal activation in in vivo cotransplanted islets with myoblast cells. (a) Protocol for the assessment of pSTAT3 expression in engrafted islets at 7 days after transplantation. (b) pSTAT3 expression in engrafted islets. Histology of engrafted islets in the islet alone group (left) and islet + myoblast group (right) at 7 days after transplantation. Green, insulin; red, pSTAT3; blue, DAPI. (c) The proportion of pSTAT3-positive area to insulin-positive area in the engrafted islets in the islet alone group (white, *n* = 5) and islet + myoblast group (black, *n* = 5). (d) Protocol for islet proliferation analysis in mice, using BrdU incorporation. Nephrectomy was performed 4 h after the last BrdU injection. (e) BrdU expression in the engrafted islets from the islet alone group (left) and islet + myoblast group (right) at 7 days after transplantation. Green, insulin; red, BrdU; blue, DAPI. (f) The proportion of BrdU-positive area to insulin-positive area in the engrafted islets from the Islet alone group (white, *n* = 5) and islet + myoblast group (black, *n* = 5). Values were compared using Student's *t*-test. Data are presented as the mean ± SEM. ^∗^*p* < 0.05 and ^∗∗^*p* < 0.01. BrdU, bromodeoxyuridine; DAPI, 406-diamidino-2-phenylindole; pSTAT3, phosphorylated signal transducer and activator of transcription 3; SEM, standard error of the mean.

Next, we investigated whether JAK-STAT signal activation—a candidate mechanism for the myoblast cell-induced enhanced islet graft function—was promoted within the in vivo removed grafts after cotransplantation of myoblast cells with islets. Immunohistochemical assessment of grafts that were removed 7 days after ITx revealed that the pSTAT3-positive cell rate was significantly higher in the islet + myoblast group than in the islet alone group (*p* = 0.0239) (Figures 7(a)–7(c)). It was previously reported that pancreatic islet proliferation is promoted through JAK-STAT signaling [31], so we also investigated islet proliferation using the BrdU incorporation assay. The ratio of BrdU-positive cells was significantly higher in the islet + myoblast group than in the islet alone group (*p* = 0.0475) (Figure 7(d)–7(f)). These data confirmed the enhancement of JAK-STAT signaling activation in in vivo islets that were cotransplanted with myoblast cells, verifying that JAK-STAT signal activation was a possible mechanism for the enhanced islet graft function by the myoblast cell cotransplantation.

### 3.7. The Fate of the Myoblast Cells After Transplantation

Finally, to evaluate the fate of myoblast cells after transplantation, we performed immunohistochemical staining for desmin as a marker of myoblast cells on grafts removed 7 and 30 days after ITx in the islet + myoblast group. Many desmin-positive cells were observed around the islet graft at 7 days, but only a few desmin-positive cells were present at 30 days (Figure S3). This result suggested that transplanted myoblast cells persisted for 7 days but then gradually declined to just a few by 30-day postimplantation.

## 4. Discussion

**Figure 8:**
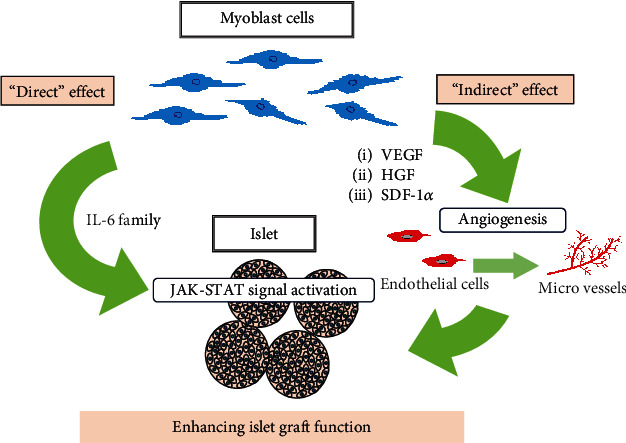
Suggested mechanism underlying the enhanced islet graft function by myoblast cell cotransplantation. The results of this study suggest two possible mechanisms underlying enhancement of islet graft function with myoblast cell cotransplantation: “indirect effects” mediated by angiogenesis induced by angiogenesis-related cytokines secreted by the myoblast cells and “direct effects” on enhanced islet graft function via JAK-STAT signal activation due to IL-6 secreted by the myoblast cells. HGF, hepatocyte growth factor; SDF-1*α*, stromal-derived factor-1*α*; VEGF, vascular endothelial growth factor.

In this study, we first performed in vitro experiments, and the results supported the possibility that myoblast cells contributed to the maintenance of differentiated islet phenotypes and enhanced the insulin secretion capacity of islet. To verify this possibility, we next performed in vivo experiments in diabetic mouse models. The in vivo results demonstrated that myoblast cells enhanced the function of transplanted islet grafts in diabetic mice. We also further investigated the mechanism underlying the enhanced islet graft function via myoblast cell cotransplantation. Our results suggested two possible mechanisms: “indirect effects” mediated by angiogenesis and “direct effects” of myoblast cells on the islets (Figure 8). With regard to the first proposed mechanism, myoblast cells reportedly exhibit the capacity to secrete angiogenesis-related cytokines, including VEGF, HGF, and SDF-1*α* [12, 15, 16]. Consistently, our present results confirmed that these cytokines were increased in the supernatants of islets cocultured with myoblast cells, compared to islets cultured alone. We also observed that the cotransplantation of myoblast cells induced significantly more CD31-positive microvessels and significantly more CD31^+^/*α*-SMA^+^ vessels. Notably, several previous studies have reported that vascularized islets exhibit enhanced insulin secretion capacity [7, 32]. These findings imply that indirect angiogenesis-mediated effects may underlie the enhanced islet graft function seen with myoblast cell cotransplantation.

The second proposed mechanism is that the cytokine IL-6, which is secreted by myoblast cells, may “directly” activate JAK-STAT signaling in the islets, resulting in enhanced function of the islets themselves. Skeletal muscle produces and releases IL-6, which is an activator of JAK-STAT signaling [33, 34]. Moreover, IL-6 receptors are expressed in islets and regulate islet proliferation [35], and several reports have demonstrated that IL-6 activates JAK-STAT signaling in islets [36, 37]. STAT3 modulates the cell cycle in islets [31], although the impact of JAK-STAT signaling on islet insulin secretory capacity is controversial [34–38]. In the present study, we showed that myoblast cells secreted IL-6 in our in vitro experiment and that myoblast cells enhanced JAK-STAT signaling in islets in both our in vitro and in vivo experiments. Furthermore, we demonstrated that addition of a JAK inhibitor significantly weakened the increased insulin secretion in islets cocultured with myoblast cells. These data support our proposal that the enhanced islet function is caused by a direct effect, in which JAK-STAT signaling is activated by IL-6 secreted by the myoblast cells.

In current clinical practice, ITx is typically performed by direct infusion of islets via the portal vein into the liver. Although islet graft destruction occurs regardless of transplant site, an estimated 60%–80% of islets are lost immediately after intraportal ITx through an instant blood-mediated inflammatory reaction [39], leading to a research focus on establishing an optimal transplant site that protects islets. Candidate sites include the intramuscular space [40–42], omentum [43], peritoneum [44], kidney capsule [26, 45], bone marrow [46], and subcutaneous spaces [25]. Of the candidates, subcutaneous sites offer the most surgical advantages because of their safety, ease of transplantation, availability for retrieval and biopsy of transplants, and large transplant volume capacities [47], However, subcutaneous sites lack the vascularity required for engraftment, leading to shortages of oxygen and nutrients [48, 49]. Therefore, it is clinically important to establish an optimal ITx method that enables successful subcutaneous transplantation.

In this study, we selected the kidney capsule as the transplant site because it is frequently used for positive controls in ITx experiments. Our present results may provide valuable information for efforts to resolve current issues regarding ITx. ITx with myoblast cells could represent a possible option for establishing an optimal ITx method at subcutaneous sites. For implanting myoblast cells with a subcutaneous ITx, myoblast cell sheets may be appropriate because they enable administration of many cells at once. In fact, several recent reports describe ITx performed at a subcutaneous site using mesenchymal stem cell sheets as a scaffold for the islets [50, 51]. Additionally, Miyake et al. have described a new method of myoblast administration using cell sheets in the context of peripheral vascular disease. In their method, cell sheets are crushed to create clustered myoblast cells, which are then administered by local injection [27]. Cell transplantation by local injection using cell sheet technology may be desirable because it does not require surgical subcutaneous dissection and can be performed along with subcutaneous ITx without destroying the subcutaneous vascular network.

Intramuscular ITx seems similar to the myoblast cell transplantation described here, but with a poor transplant efficacy in previous reports [42], indicating divergence from our results. We have not investigated this discrepancy, but considering the small population of myoblast cells in muscle, the discrepancy could trace to differences between the two approaches in the numbers of transplanted myoblast cells.

Another important issue to consider is the myoblast cell donor. After muscle specimen harvest, myoblast cells must be cultured for about a month to reach sufficient numbers for transplantation. Thus, these cells would likely need to be prepared before islets, so that isolating both cell types from the same donor simultaneously would be untenable. Kenyon et al. demonstrated longer islet allograft survival with autologous mesenchymal stem cells than with donor or third-party mesenchymal stem cells; with the immune rejection induced by ITx, better results would be expected using autologous myoblast cells [52]. Taken together, these findings indicate that use of autologous myoblast cells may be preferable over using donor or third-party-derived myoblast cells, despite the additional surgical invasiveness involved in harvesting muscle.

This study has several limitations. First, the transplantation experiment was conducted for a short observation period of 1 month, so that the long-term effects could not be evaluated. Another limitation is that we did not characterize the optimal conditions under which myoblast cells most effectively enhanced islet graft function. Further studies are needed for optimization of these effects.

In conclusion, myoblast cells enhanced the function of transplanted islet grafts in diabetic mice. Based on our in vitro and in vivo findings, we propose two possible mechanisms for this effect: angiogenesis and enhanced islet graft function due to JAK-STAT signal activation. The present results suggest the possible clinical utility of including myoblast cells in ITx. Further studies, such as subcutaneous ITx experiments with myoblast cells, will be required to develop the clinical application of myoblast cells in this setting.

## Data Availability

The datasets generated and/or analyzed during the current study are available from the corresponding author on reasonable request.

## Supporting Information

Supporting Information Additional supporting information can be found online in the Supporting Information section. Figure S1 Nonfasting blood glucose levels in individual animals after transplantation. Figure S2 RNA sequencing revealed upregulation of the JAK-STAT cascade in islets cocultured with myoblast cells. Figure S3 Desmin expression around engrafted islets. Table S1 Primers used for quantitative real-time polymerase chain reaction.
