# Supplementary material for: Skeletal Myoblast Cells Enhance the Function of Transplanted Islets in Diabetic Mice
Source: J Diabetes Res. 2024 May 17;2024:5574968. doi: 10.1155/2024/5574968 (PMC11126349; doi:10.1155/2024/5574968)
Supplement: Supporting Information — Additional supporting information can be found online in the Supporting Information section. Figure S1 Nonfasting blood glucose levels in individual animals after transplantation. Figure S2 RNA sequencing revealed upregulation of the JAK-STAT cascade in islets cocultured with myoblast cells. Figure S3 Desmin expression around engrafted islets. Table S1 Primers used for quantitative real-time polymerase chain reaction. [file 5574968.f1.docx]

**Supplemental Digital Content**


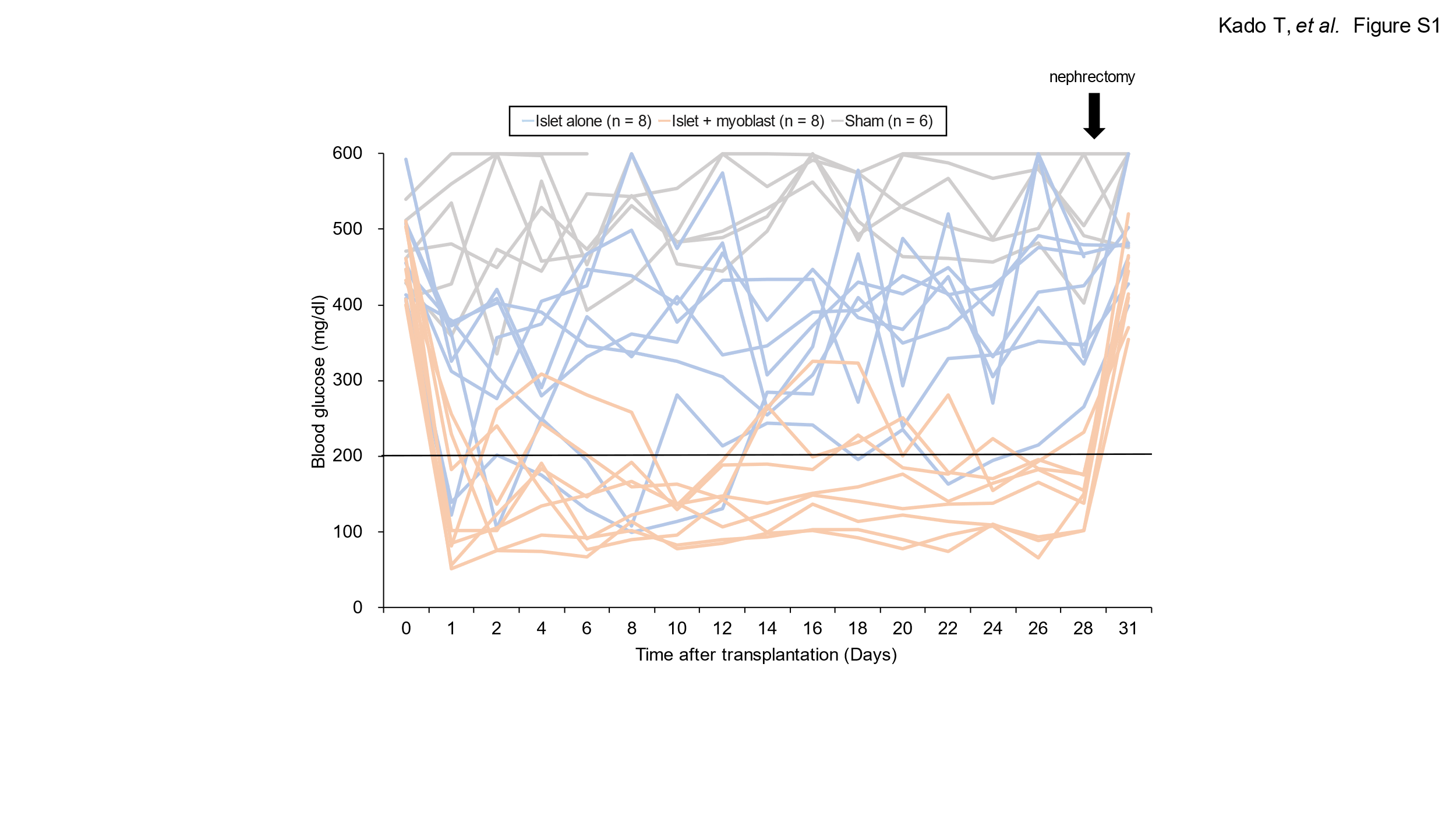


**Figure S1. Non-fasting blood glucose levels in individual animals after transplantation**

Individual non-fasting blood glucose levels after transplantation in the Islet alone group (blue, n = 8), Islet + myoblast group (orange, n = 8), and Sham group (grey, n = 6). At 30 days after transplantation, the kidney was removed (arrow).


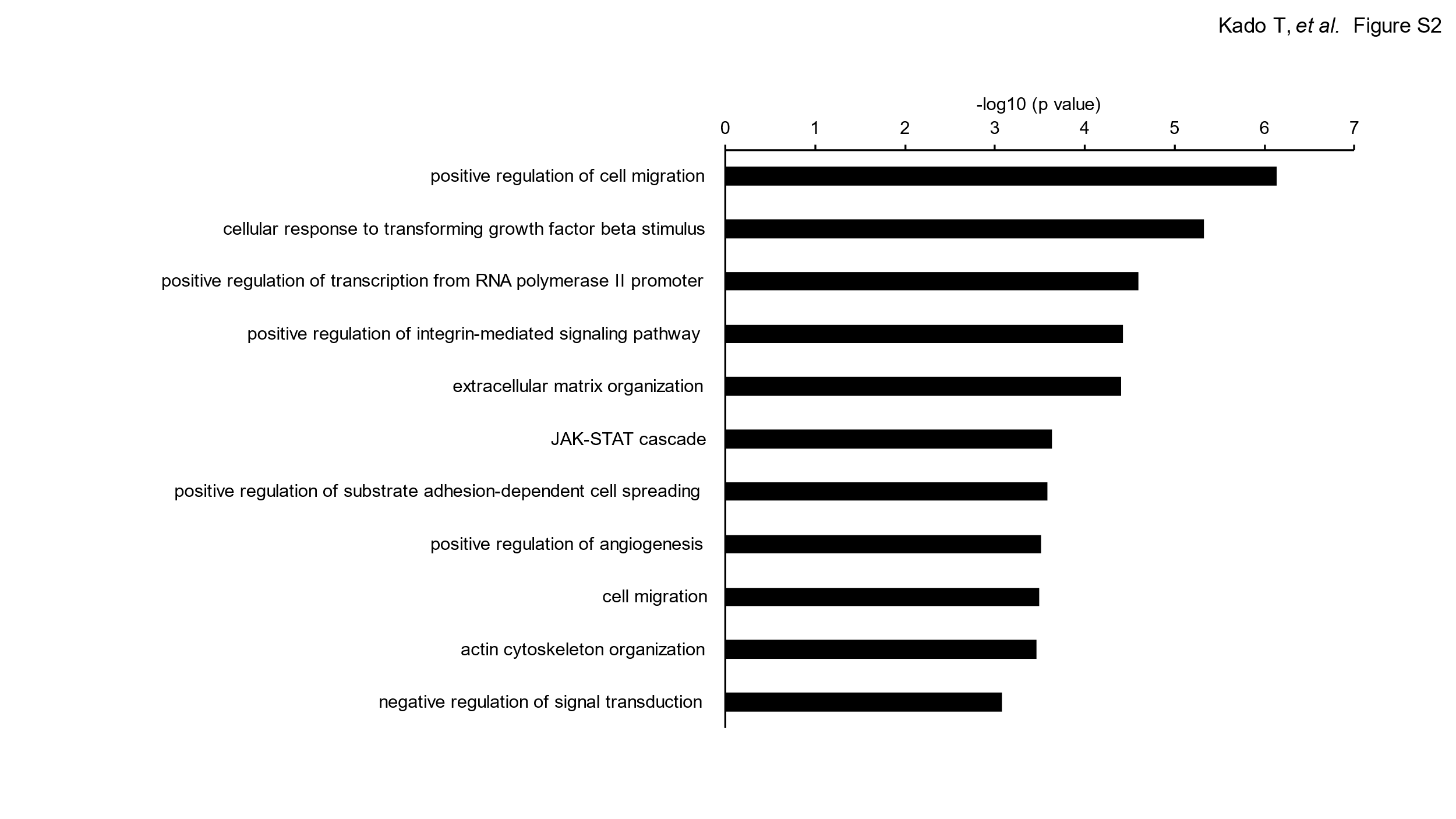


**Figure S2. RNA sequencing revealed upregulation of the JAK-STAT cascade in islets co-cultured with myoblast cells.**

Enrichment analysis of differentially expressed genes from islets after 24 h of co-culture with myoblast cells, using the terms of the Gene Ontology biological process (adjusted p values < 0.001). Data show different regulation among islets cultured with myoblast cells compared to islets cultured alone.


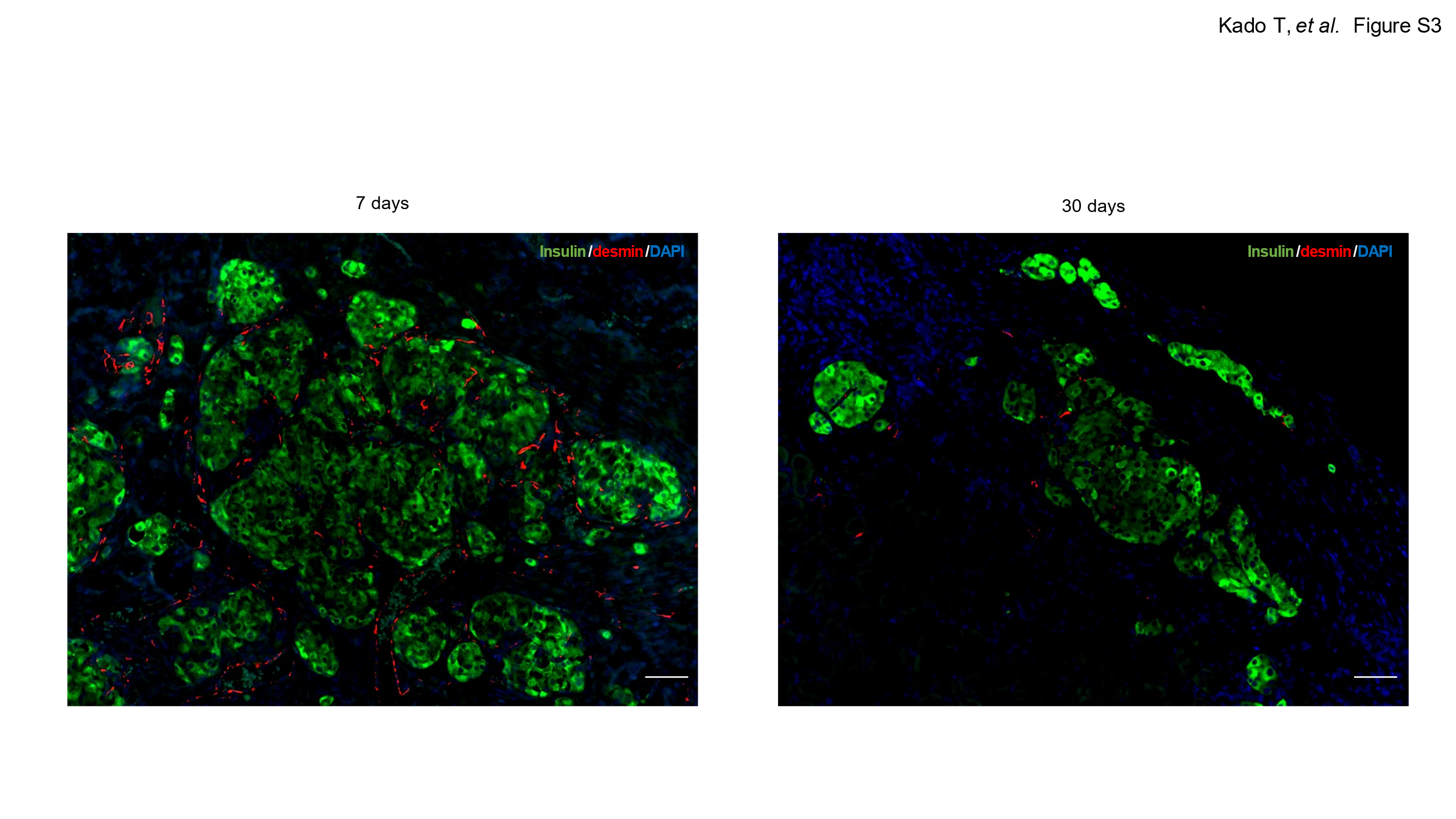
 **Figure S3. Desmin expression around engrafted islets.**

Histology of engrafted islets from the Islet + myoblast group at 7 days (left) and 30 days (right) after transplantation. Green, insulin; red, desmin; blue, DAPI. Scale bars: 50 µm. DAPI, 406-diamidino-2-phenylindole.

**Table S1. Primers used for quantitative real-time polymerase chain reaction**

| Genes | Sequences (5ʹ to 3ʹ) | |
| --- | --- | --- |
|  | Forward primer | Reverse primer |
| *ins2* | AACATGGCCCTGTGGATGCG | ACCCAGCTCCAGTTGTGCCA |
| *pdx1* | CGGACATCTCCCCATACG | AAAGGGAGCTGGACGCGG |
| *neurog3* | TGCAGCCACATCAAACTCTC | GGTCACCCTGGAAAAAGTGA |
| *mki67* | CCAGCACTCCAAAGAAACCC | ATTTTGTAGGGTCGGGCAGG |
| *pbk* | AGAAGCTTGGCTTTGGGACT | TTCACGGCCCAAGGAGAATG |
| *cdk1* | CTGCAGCTCGGAGCACAGTT | CCAGAACACGGAGGCACTTG |
| *gapdh* | TGCCCCCATGTTTGTGATG | TGTGGTCATGAGCCCTTCC |

cdk1, cyclin dependent kinase 1; gapdh, glyceraldehyde-3-phosphate dehydrogenase; ins2, insulin 2; mki67, marker of proliferation Ki-67; neurog3, neurogenin 3; pbk, PDZ binding kinase; pdx1, pancreatic and duodenal homeobox 1.
